# Immediate Postabortion Family Planning Uptake and Its Associated Factors Among Women Seeking Abortion Services at Health Facilities in East Shewa Zone, Ethiopia: A Multicenter Cross-Sectional Study

**DOI:** 10.1155/tswj/7915677

**Published:** 2025-10-14

**Authors:** Mangistu Abera, Mulugeta Animaw, Abdulaziz Assefa, Ayele Sahile Abdo, Aynalem Belay, Fikremariam Endeshaw, Daniel Tsega, Aberash Beyene Derribow

**Affiliations:** Department of Midwifery, College of Medicine and Health Sciences, Wolkite University, Wolkite, Ethiopia

**Keywords:** contraceptive method, Ethiopia, family planning, PAFP uptake, postabortion

## Abstract

**Background:**

Immediate postabortion family planning uptake refers to the initiation and use of contraceptive methods immediately after an abortion treatment to prevent subsequent unintended pregnancy. Women should wait at least 6 months after having an abortion before becoming pregnant again, according to WHO recommendations, even if they are eager to have a child immediately. Despite this evidence, many postabortion clients leave healthcare institutions without receiving family planning advice or services. Thus, this study was aimed at assessing the immediate postabortion family planning uptake and associated factors among women seeking abortion services.

**Method:**

An institution-based, multicenter cross-sectional study was conducted among 402 women seeking abortion services in East Shewa Zone healthcare facilities from March 20 to May 25, 2022. A systematic sampling technique was employed to get a representative sample. Data were entered into EpiData Version 4.1 and then exported to Statistical Package for Social Science Version 26 for analysis. Bivariate and multivariable analyses were done to identify variables associated with the level of immediate postabortion family planning uptake in the binary logistic regression model. Statistical significance was declared at *p* value < 0.05. Finally, tables, graphs, and narration were used to present the findings.

**Result:**

In this study, the overall immediate postabortion contraceptive uptake was 70.1% (95% CI: 65.4, 74.6). Being single (AOR = 4.0; 95% CI: 1.8, 8.0), educational status (AOR = 3.7; 95% CI: 1.4, 9.7), service received at public facility (AOR = 3.0; 95% CI: 1.5, 6.1), previous information about family planning (AOR = 2.1; 95% CI: 1.2, 3.9), previous use of contraceptive method (AOR = 5.4; 95% CI: 2.9, 9.9), and postabortion family planning counseling (AOR = 5.7; 95% CI: 3.1, 10.4) were significantly associated with immediate postabortion family planning uptake.

**Conclusion and Recommendation:**

In this study, 29.9% lacked postabortion family planning uptake. Being single, educational status, service received at a public facility, previous information about family planning, previous use of a contraceptive method, and postabortion family planning counseling showed significant associations with immediate postabortion contraceptive uptake. Therefore, it is necessary to establish effective educational awareness and counseling aimed at promoting postabortion modern family planning uptake among the abortion service-seeking women.

## 1. Introduction

Immediate postabortion family planning (PAFP) uptake is the initiation and use of family planning methods immediately or within 48 h [1, 2]. Using PAFP reduces unintended pregnancies and the recurrence of abortions [3, 4]. It also reduces the risks of adverse maternal and perinatal outcomes for pregnancies following induced abortion [5].

Globally, 44% of pregnancies were unintended, and two-fifths of these pregnancies [6] ended by abortion [7]. Around three out of four women left the health facilities without a contraceptive method after receiving abortion care globally [4, 8]. Further, due to the nonuse of contraception, more than 40% of pregnancies are unplanned globally [1]. The World Health Organization (WHO) estimates that every year, nearly 5.5 million African women have an unsafe abortion. In Eastern Africa, it is estimated that 18% of all maternal deaths are the result of complications of poorly performed abortions [9]. Fifty-five million unintended pregnancies in developing countries occur every year due to women not using contraceptives [3, 10].

The nationally representative study in Ethiopia, conducted in 2014, also found that 25% of facilities in the country were not suitable for providing postabortion contraceptive services for women who had received abortion care, and one-fifth did not receive immediate PAFP services after seeking abortion [11]. These put women at increased risk of another unwanted pregnancy and possibly ending in another abortion [12]. Women who have had an abortion have had a previous abortion most of the time [4], yet many of these women did not have access to contraceptives and did not get immediate PAFP services [13].

Following an abortion, contraception is recommended to prevent unintended pregnancies and repeat abortions due to adverse health consequences like placenta previa, ectopic pregnancy, preterm birth, subfertility, and breast cancer, recommending modern, long-acting contraceptive utilization [3, 14]. For many postabortion patients, the lack of family planning counseling and services quickly leads to another abortion [3, 15]. This makes it essential to ensure that PAFP counseling and service delivery are offered to all women who are present for emergency obstetric or postabortion care, regardless of the method of treatment [3].

To achieve Sustainable Development Goal 3, reducing maternal mortality by providing postabortion contraceptive services to ensure good health and well-being is crucial [16]. Ethiopia's comprehensive strategy for abortion care and the implementation of a new health sector transformation plan (HSTP) aim to improve maternal healthcare services postabortion, as low utilization rates result from the lack of contraceptive services, leading to high maternal mortality rates [17]. The new plan is aimed at improving access to information and methods for PAFP uptake. To the knowledge of the investigator, no evidence indicates the immediate PAFP uptake and associated factors, particularly in the study area. In Ethiopia, PAFP uptake is difficult due to a lack of knowledge, fear of adverse effects, social pressures, and restricted access to treatments [18] Therefore, this study was aimed at assessing the immediate PAFP uptake and associated factors among women seeking abortion services at healthcare facilities in East Shewa Zone, Ethiopia.

## 2. Materials and Methods

### 2.1. Study Area and Period

The study was conducted in East Shewa Zone, Ziway Dugda Woreda, from March 20 to May 25, 2022. Ziway Dugda Woreda is located in the Oromia regional state. It is about 165 km away from the capital city of Ethiopia, Addis Ababa. The woreda is located in the middle of the Ethiopian Rift Valley, with a total population increasing from 120,862 in 2007 to 196,678 (100,761 males and 95,917 females), with a 45,080 estimated reproductive age group based on Ethiopian projections for 2017 due to the flourishing of industries such as floriculture and agroprocessing industrial parks. The woreda has two hospitals (one government and one NGO) and six government health centers. These health institutions provided comprehensive abortion and family planning services under IPAS support. Within 1 year, around 2940 postabortion clients visited the health institutions, and on average, 30 women received abortion services in 1 month, and 20 health professionals took PAFP training.

### 2.2. Study Design and Population

A multicenter facility–based cross-sectional study design was conducted from March 20 to May 25, 2022. The source population comprises all women who receive abortion services in health institutions in Ziway Dugda Woreda. All women who received abortion services during the study period were included in the study. Postaborted women who were acutely and critically ill and needed a referral for immediate treatment were excluded from the study.

### 2.3. Sample Size Determination

The sample size was determined by using a single population proportion formula based on assumptions of frequency (prevalence of PAFP utilization of 61% from a study conducted in Bahir Dar [19], 95% CI, 5% margin of error, and 10% nonresponse rate), which yielded 402. Sample size determination for the second objective: the factors for PAFP obtained from the same study and calculated by Epi Info 7 menu statically, with assumptions of a 95% CI, 80% power, and an exposure to the unexposed ratio of 1:1. Since the sample size for a single population proportion (402) was greater than the sample for associated factors (130, 65, and 71), we got a final sample size of 402.

### 2.4. Sampling Techniques and Procedure

**Figure 1:**
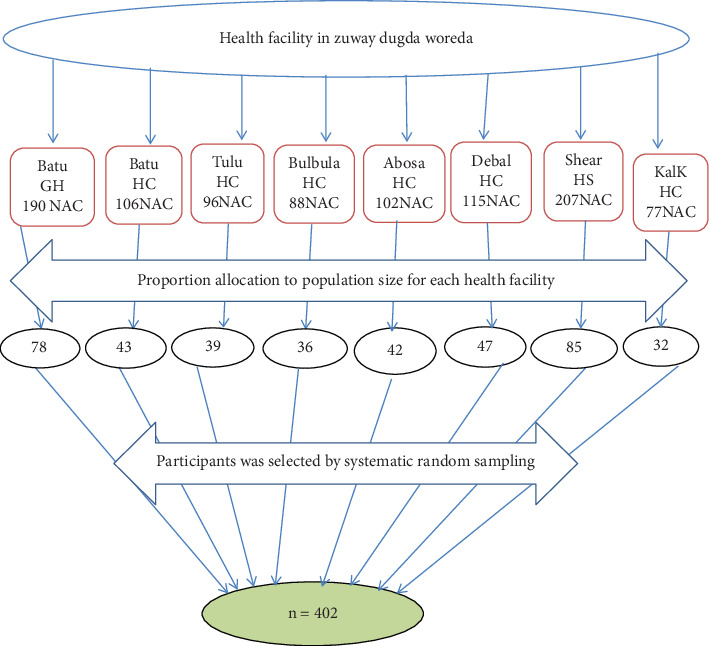
Schematic presentation of sampling procedure of immediate postabortion family planning uptake and associated factors in Ziway Dugda Woreda, 2022 (*n* = 402).

All eight health institutions that provide abortion services were included in Ziway Dugda Woreda. On average, among all those health institutions, there were 981 (*N*) abortion service users per month. Then, the possible number of respondents in each of the health institutions was allocated proportionally based on the per-month average number of clients flowing for abortion services. Then, systematic random sampling was used to select the study participants in each healthcare facility as *K* = *N*/*n*, where *K* is the skipping interval and is three. The woman who came for the abortion service on the first day of data collection was considered the first respondent. Then, each respondent corresponding to the skip interval was selected (Figure 1).

### 2.5. Study Variables

*Dependent variable*: immediate PAFP uptake.

*Independent variables*: sociodemographic characteristics such as age, religion, residence, marital status, educational level, spouse's educational level, and occupation.

*Reproductive health factors*: gravidity, parity, number of alive children, previous abortion history, condition of current pregnancy, type of abortion, gestational age, and FP use history.

Health service–related factors include PAFP counseling, the owner of the facility, the type of management, and the method of choice. Personal factors include family planning information, the source of information, and the reason for terminating the current pregnancy.

### 2.6. Operational Definitions

Abortion is the expulsion of the fetus from the uterus or termination of pregnancy before 28 completed weeks of gestation [11, 20].

*PAFP utilization*: the use of modern family planning methods among clients who receive abortion care in the health facility [18].

*Immediate PAFP uptake*: immediate PAFP uptake refers to the initiation and use of contraceptive methods immediately or within 48 h after an abortion treatment to prevent subsequent unintended pregnancy [1, 2, 21, 22].

### 2.7. Data Collection Tool and Data Collection Procedures

A structured interviewer-administered questionnaire was developed after reviewing the relevant literature and used for data collection [1, 2, 21, 22]. The questionnaires were then prepared in English, translated into Amharic and Afan Oromo languages, and then translated to English to check for any inconsistencies. This research utilized a structured questionnaire that underwent a pretest to ensure clarity, appropriateness, and reliability. The tool's content validity was checked by a panel of experts for relevance and essentiality, and its content reliability was assessed using Cronbach's alpha, resulting in a 0.89 Cronbach's alpha value for PAFP uptake-related questionnaires.

Eight BSC nurses were assigned for data collection, and two supervisors. The questionnaire contained sociodemographic characteristics, previous reproductive health history, personal factors, and a PAFP uptake question. All the participants were reached through exit interviews in a private place after the women received abortion services. The interviewers collected data after obtaining written informed consent from the study participants. The completeness of the questionnaire was regularly checked, and finally, the reviewed questionnaires were returned to the principal investigator for further cross-check.

### 2.8. Data Quality Management

To maintain the data quality, the questionnaire was prepared first in English, then translated into the local language by an independent language expert to ensure its consistency. The principal investigator gave training to both data collectors and supervisors. The questionnaire was pretested on 5% of the sample size in the Mojo health center. Based on the results obtained, the necessary modifications were made. The data collectors examined the completeness of the data during data collection, and the supervisor and lead investigator examined it immediately after data collection. Data were reviewed, coded, entered, and cross-checked during data entry into the computer software before analysis.

### 2.9. Data Analysis Procedures

Data entry was done using EpiData Version 4.1. The entered data was checked and exported to SPSS Version 26 for data analysis. Different frequency tables, graphs, and descriptive summaries were used to describe the study variables. In bivariate analysis, a crude odds ratio (COR) with 95% CI was used to identify the candidate variables for multivariable analysis by using the binary logistic regression model. The result was presented as a COR to show the strength of the association between the independent variable and the dependent variable. Independent variables with a significance level of *p* value < 0.25 at 95% CI in bivariable analysis and which were fit for the regression model were retained for inclusion into the multivariable logistic regression to control all possible confounders.

Multicollinearity was checked to see the linear correlation among the associated independent variables by using the variance inflation factor (VIF) and standard error. A VIF of > 10 or a standard error of > 2 was considered suggestive of multicollinearity. No multicollinearity was detected during the analysis. For all independent variables, the multicollinearity effect was checked by collinearity diagnostic statistics via VIFs and tolerance tests with a maximum value of 2.36 and a minimum value of 35.7%, respectively.

In multivariable analysis, the multivariable logistic regression model was used to control the confounders. The Hosmer–Lemeshow goodness-of-fit test was done to check for model fitness with a *p* value of 0.646, which indicates the model was fitted. Adjusted odds ratio (AOR) with a 95% confidence interval was estimated to demonstrate the strength of the association between the independent variable, after controlling for the effects of confounders. The results were considered statistically significant at a *p* value < 0.05. Finally, tables, graphs, and narration were used to present the findings.

## 3. Results

A total of 402 study participants were interviewed and gave a response rate of 100%. The results were presented as follows: 402 study participants were interviewed and gave a response rate of 100%, and the results were presented.

### 3.1. Sociodemographic Characteristics of the Study Participants

About 149 (37.1%) women were in the 20–24 age group, with a mean age and SD of 24.7 ± 8.3 years, and 163 (40.5%) respondents were followers of the Muslim religion. Then, 261 (64.9%) of respondents lived in a rural area, and nearly three-quarters (71.4%) were married. Then, 129 (32.1%) completed primary education (Table 1).

### 3.2. Reproductive Health Characteristics of the Study Participants

The study revealed that from the total respondents, about 249 (61.9%) of respondents were gravida 2–4, 146 (36.3%) of the participants were nulliparous, and about 230 (57.2%) of the study respondents had 1–3 alive children. Contraceptive history before the latest index of pregnancy information was assessed: 231 (57.5%) used FP previously, only 63 (15.7%) had a previous abortion history, and more than three-quarters of 52 (81.2%) of them faced abortion at least once (Table 2).

### 3.3. Immediate PAFP Uptake of Study Participants

**Figure 2:**
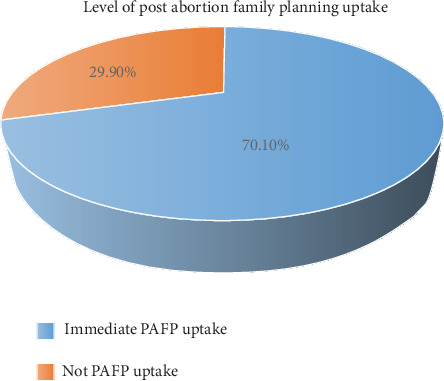
The level of immediate postabortion family planning uptake among women seeking abortion services in East Shewa Zone, Ethiopia, 2022 (*n* = 402).

Out of 402 study participants, 282 (70.1%) have had immediate contraceptive uptake after they received postabortion care services (Figure 2).

Among those who used contraceptives, more than half (63%) used injection methods. Then, 317 (78.9%) of study participants received abortion services in public health facilities, whereas 85 (21.1%) received abortion services in private clinics. Before leaving the health facility, only 275 (68.4%) of respondents received PAFP counseling.

### 3.4. Factors Associated With Immediate PAFP Uptake

Bivariate logistic regression analysis showed that age, marital status, educational level, residence, occupation, gravidity, number of alive children, ownership of the facility, information access, ever-used family planning, and PAFP counseling were found to be candidate variables at a *p* value of 0.25 for the multivariable analysis. In multivariable analysis, marital status, educational level, ownership of the facility, information access, having used previous contraceptives, and PAFP counseling were factors that affected immediate PAFP uptake at a *p* value of less than 0.05. In this study, single women were four times (AOR = 4.0; 95% CI: 1.8, 8.0) more likely to utilize PAFP than married women. Women who attended secondary education were 3.7 times (AOR = 3.7; 95% CI: 1.4, 9.7) more likely to use PAFP when compared with those women who did not attend formal education. Women who got abortion services at public health facilities were three times more likely to utilize PAFP as compared to those who got abortion care services in private clinics (AOR = 3.0; 95% CI: 1.5, 6.1). Those respondents who had previous information about PAFP were 2.1 times (AOR = 2.1; 95% CI: 1.2, 3.9) more likely to use PAFP compared with those women who did not have previous information. Similarly, women who used the previous contraceptive were 5.4 times more likely to utilize contraceptives as compared to their counterparts (AOR = 5.4; 95% CI: 2.9, 9.9). Women who received family planning counseling during service provision were 5.7 times (AOR = 5.7; 95% CI: 3.1, 10.4) more likely to accept PAFP compared with women who did not uptake the immediate PAFP (Table 3).

## 4. Discussion

Immediate PAFP uptake is critical for women's health. Thus, lack of immediate PAFP is the root cause of major subsequent unwanted pregnancies, which are associated with maternal morbidity and mortality. This facility-based cross-sectional study identifies factors associated with immediate PAFP uptake in the study settings. Marital status, educational level, service received facility, information access, having used previous contraceptives, and PAFP counseling were all associated with immediate PAFP uptake.

In this study, 70.1% (95% CI: 65.4, 74.6) of women had uptake of family planning after receiving abortion care. This study was consistent with previous studies conducted in Jimma (70.1%) and Gambella (72.9%) [23, 24], respectively. This similarity might be due to the relatedness of participants' socioeconomic status; in all studies, participants were from developing countries, and it could also be due to study design and sample size consistency. This study was higher compared to the previous study done in Bahir Dar town (61%), Assela (53.7%), Shire town (61.5%), and Dessie Town (47.5%) [25–28]. The difference might be due to sample size differences, study year interval, and the number of respondents who have information about family planning. The possible reasons could be that information may change their beliefs, perceptions, misperceptions, rumors, and practices [29, 30]. The other possible reasons might be insufficient counseling, a desire to have more children, the judgmental approach of healthcare providers, and misconceived rumors about family planning methods [30]. On the other hand, this study is lower than the study conducted in Addis Ababa (86%) [31]. The difference could be due to the education level. Individuals who are educated have a better chance of accessing reproductive health information, and education helps them understand their reproductive and sexual rights and responsibilities. It also enables them to discuss with their partners and make joint decisions on family planning and family size [32, 33]. Also, the current finding was lower than the studies done in Brazil (97.4%) [34] and Dar es Salaam, Tanzania (89%) [35]. The probable differences might be due to the study setup, cultural differences, and sociodemographic characteristics of the respondents. In addition, in Tanzania, the study design was a cohort, which differed from the current study design.

Single women were four times more likely to uptake PAFP as compared to married women. This aligns with a study conducted in Debre Markos [36] and Gambella [23]. The possible reason could be that unmarried women have a strong desire to use family planning due to fear of pregnancy because of cultural outcasts and social discrimination [34]. Women who attended above-secondary education were 3.7 times more likely to uptake PAFP compared with those women who did not attend formal education. The findings of this study were similar to the previous studies conducted in Gambella [23], Bahir Dar town [26], as well as the study in Pakistan [37]. This could be due to being more concerned about their career development and better contraceptive knowledge [38, 39]. In addition, they increase their chances of accessing reproductive health information. Also, better education might have been associated with increased women's income and social independence, where they are less likely to be influenced by social norms in making fertility and contraception decisions [40, 41]. In this finding, women who received services in public health facilities were three times more likely to uptake PAFP as compared with services received in private facilities. This finding was supported by a study done in Tigray town [18]. This might be due to the current situation in Ethiopia: family planning services are provided free of charge in all public health facilities, whereas the services in private facilities have a cost [18]. Those respondents who had gotten information about PAFP before coming to the abortion service were 2.1 times more likely to accept PAFP uptake compared with those who had not gotten information before. This finding is supported by the study done in Bahir Dar [42] and Kenya [43], respectively. This is due to the evidence that information systems are one of the elements of successful PAFP programs [44]. Furthermore, women who had gotten information about family planning improved their knowledge and increased their self-efficacy or empowerment in making better decisions regarding family planning use. In addition, those who had information may change their beliefs, perceptions, misperceptions, and practices [45]. Women who had a previous history of contraceptive utilization were 5.4 times more likely to uptake PAFP as compared to their counterparts. This study aligns with studies done in the Shire [25], Bahir Dar [19], and Pakistan [37]. This might be because previous exposure to family planning services might influence awareness and a better understanding of the different types of family planning methods, along with the advantages and disadvantages, which will improve their decision-making skills toward PAFP utilization [25]. The study showed that women who had a previous history of contraceptive use have a good attitude toward the utilization of PAFP [46].

In this study, those women who had received PAFP counseling were 5.7 times more likely to use PAFP than those who had not received PAFP counseling. This finding is consistent with the studies conducted in Debre Markos [36], Assela [28], and Shire Town [25]. The possible explanation might be that counseling helps women make informed decisions about the uptake of family planning services. In addition, counseling is one of the critical elements in the provision of quality family planning services. Counseling provision by healthcare providers is essential, which helps clients make and carry out their own choices about reproductive health and family planning [47, 48].

### 4.1. Strengths and Limitations of the Study

#### 4.1.1. Strength of the Study

The strength of this study includes various categories of women, and the sample was taken at random. Hence, the results of this study can represent the population. This study minimized potential biases by using clear objectives, pretested questionnaires, training provided for data collectors and supervisors, a random sampling method, an ideal sample size, and statistical adjustments (multivariable regression) to account for confounding variables. Finally, ethical guidelines were implemented to ensure unbiased participants.

#### 4.1.2. Limitations of the Study

This study was based on self-reported survey data, which may be influenced by recall bias and potential bias from social desirability since women may report more acceptable responses. Since a cross-sectional design was used, the cause-and-effect relationship could not be established.

## 5. Conclusion and Recommendations

In this study, 29.9% lacked PAFP uptake. Being single, educational status, service at a public facility, previous information about family planning, previous use of contraceptive methods, and PAFP counseling showed a significant association with immediate postabortion contraceptive uptake. The zonal health office should raise community awareness about immediate PAFP uptake, women's empowerment, and strengthen postabortion care services in private facilities. Healthcare providers who provide abortion services are encouraged to provide detailed PAFP counseling on fertility return to reduce unintended pregnancy by immediate PAFP uptake.

Therefore, educating women helps them overcome barriers to using PAFP. In addition, counseling on family planning is a unique opportunity to provide information about family planning and its widespread health benefits for women, facilitating decision-making for use.

## Figures and Tables

**Table 1:** Sociodemographic characteristics of the study participants in Ziway Dugda Woreda health facilities, East Shewa, Ethiopia, 2022 (*n* = 402).

**Characteristics**	**Category**	**Frequency**	**Percent**
Age (years)	15–19	66	16.4
	20–24	149	37.1
	25–29	110	27.4
	30–34	51	12.7
	≥ 35	26	6.4

Religions	Orthodox	152	37.8
	Muslim	163	40.5
	Protestant	73	18.2
	Catholic	10	2.5
	Others^a^	4	1

Marital status	Married	287	71.4
	Single	115	28.6

Residence	Urban	261	64.9
	Rural	141	35.1

Educational attainment	No formal education	88	21.9
	Primary education	129	32.1
	Secondary education	112	27.9
	Above secondary education	73	18.1

Occupation	Student	60	14.9
	Employed	117	29.2
	Housewife	109	27.1
	Farmer	64	15.9
	Others^b^	52	12.9

Husband educational attainment	No formal education	55	19.2
	Primary education	95	33.1
	Secondary education	54	18.8
	Above secondary education	79	27.5
	Do not know	4	1.4

^a^Wakefeta and Jova.

^b^No job and merchant.

**Table 2:** Reproductive health-related characteristics of respondents in Ziway Dugda Woreda health facilities, East Shewa, Ethiopia, 2022 (*n* = 402).

**Variable**	**Category**	**Frequency**	**Percent**
Gravidity	1	123	30.6
	2–4	249	61.9
	5 and above	30	7.5

Parity	Null	146	36.3
	1	109	27.2
	2–4	132	32.8
	≥ 5	15	3.7

Children alive	0	151	37.6
	1–3	230	57.2
	4 and more	21	5.2

Previous history of abortion	No	339	84.3
	Yes	63	15.7

Number of abortions	One	53	82.8
	Two	8	12.5
	Three and more	2	4.7

Previous history of PAFP use	No	20	31.2
	Yes	43	68.8

Current pregnancy intention	Wanted	298	74.1
	Unwanted	104	25.9

Type of abortion	Spontaneous	306	76.1
	Induced	96	23.9

Reason for termination of pregnancy	Fetal condition	14	3.5
	Maternal condition	14	3.5
	Rape	36	9
	Others	32	8
	Spontaneous	306	76

Methods of abortion management	Medication	141	35.1
	MVA	204	50.7
	Mixed procedures (medication and MVA)	57	14.2

Gestational age at abortion performed	< 9 weeks	196	48.8
	9–12 weeks	150	37.3
	13–28 weeks	56	13.9

Information about family planning methods	Yes	264	65.7
	No	138	34.3

Source of information	Mass media	43	16.0
	Neighbor	42	16
	Health professions	142	54
	Relative/friends	37	14.0

When to use PAFP	Immediately	282	70.2
	When it comes to my mind	64	15.9
	Do not want	33	8.2
	Do not know	23	5.7

When you plan to have another child	Within 1 year	99	24.6
	Within 2 years	105	26.1
	Three and above	85	21.1
	Do not know	101	25.1
	Do not want	12	3.1

Ownership of the facility	Public	317	78.9
	Private	85	21.1

Have you ever used contraceptives	Yes	231	57.5
	No	171	42.5

Abbreviations: MVA, manual vacuum aspiration; PAFP, postabortion family planning.

**Table 3:** Factors associated with immediate postabortion family planning uptake in Ziway Woreda health facilities, East Shewa, Ethiopia, 2022 (*n* = 402).

**Variables**	**IPAFP uptake**		**COR (95% CI)**	**AOR (95% CI)**	**p** **value**
	**Yes**	**No**			
Age					
≥ 35	15	11	0.4 (0.1, 0.9)	3.1 (0.8, 13)	0.117
30–34	33	18	0.5 (0.2, 1.1)	0.9 (0.3, 3.3)	0.960
25–29	72	38	0.5 (0.3, 1.0)	0.9 (0.13, 2.5)	0.823
20–24	110	39	0.8 (0.4, 1.5)	1.9 (0.7, 5.5)	0.224
15–19	52	14	1	1	
Marital status					
Single	99	16	3.5 (2.0, 6.3)	4.0 (1.8, 8.0)	0.001
Married	183	104	1	1	
Educational attainment					
Above secondary education	62	11	7.1 (3.3, 15.2)	3.7 (1.4, 9.7)	0.007
Secondary education	93	19	6.2 (3.2, 11.8)	2.0 (0.9, 4.5)	0.089
Primary education	88	41	2.7 (1.5, 4.7)	1.8 (0.9, 3.8)	0.119
No formal education	39	49	1	1	
Residence					
Urban	200	61	2.4 (1.5, 3.7)	1.0 (0.5, 2.0)	0.890
Rural	82	59	1	1	
Occupation					
Student	53	7	1	1	
Employed	86	31	0.4 (0.2, 0.9)	0.4 (0.1, 1.6)	0.184
Housewife	76	33	0.3 (0.1, 0.7)	0.5 (0.1, 2.2)	0.367
Farmer	32	32	0.1 (0.1, 0.3)	0.7 (0.1, 3.4)	0.629
Others	35	17	0.3 (0.1, 0.7)	0.6 (0.1, 2.6)	0.501
Gravida					
5 and above	15	15	0.3 (0.1, 0.8)	3.0 (0.6, 15.4)	0.185
2–4	175	74	0.8 (0.5, 1.3)	3.0 (1.0, 8.6)	0.043
1	92	31	1	1	
Children alive					
4 and above	9	12	0.2 (0.1, 0.6)	1.8 (0.2, 17.9)	0.603
1–3	71	159	0.7 (0.5, 1.2)	0.6 (0.2, 2.2)	0.421
Null	114	37	1	1	
Ownership of the facility					
Public	246	71	4.7 (2.8, 7.8)	3.0 (1.5, 6.1)	0.002
Private	36	49	1	1	
Information about PAFP					
Yes	214	50	4.4 (2.8, 6.9)	2.1 (1.2, 3.9)	0.013
No	68	70	1		
Ever used contraceptives					
Yes	202	29	7.9 (4.8, 13.0)	5.4 (2.9, 9.9)	< 0.0001
No	80	91	1	1	
Postabortion family planning counseling					
Yes	230	45	7.4 (4.6, 15.0)	5.7 (3.1, 10.4)	< 0.0001
No	52	75	1	1	

Abbreviations: AOR, adjusted odds ratio; CI, confidence interval; COR, crude odds ratio; IPAFP, immediate postabortion family planning; PAFP, postabortion family planning.

## Data Availability

The data that support the findings are available from the corresponding author upon reasonable request.

## Nomenclature

CI: confidence interval
IUD: intrauterine device
LMIC: low and middle-income countries
NGO: nongovernmental organization
OR: odds ratio
PAC: postabortion care
IPAFP: immediate postabortion family planning
SPSS: Statistical Package for the Social Sciences
